# Perceptions of the usefulness of external support to immunization coverage in Chad: an analysis of the GAVI-Alliance cash-based support

**DOI:** 10.11604/pamj.2013.15.44.2006

**Published:** 2013-06-07

**Authors:** Paulo Ferrinho, Mohammed Dramé, Prosper Tumusiime

**Affiliations:** 1 Department of International Public Health and Biostatistics, WHO Collaborating Centre for Health Workforce Policy and Planning, CMDT, Instituto de Higiene e Medicina Tropical, Universidade Nova de Lisboa. 1349-008, Lisbon, Portugal; 2 Department of Health Systems Policies and Workforce, WHO, Geneva; 3 WHO Inter-country Support Team/ESA, Harare, Zimbabwe

**Keywords:** Africa, Chad, global health initiatives, immunization, vaccination coverage

## Abstract

**Introduction:**

Chad is one of the countries supported by the GAVI-Alliance that remains with unsatisfactory vaccination coverage. This paper tries to understand the main barriers to better coverage.

**Methods:**

These barriers were categorised as up or downstream against the health system building blocks as proposed by WHO and compared with barriers and activities identified by the country in its health system's strengthening grant proposal as approved by the GAVI Alliance in 2007. Data were collected using a modified Delphi system and by analysis of grant and annual report documents.

**Results:**

Most of the activities anticipated under the GAVI health system's strengthening proposal are activities targeting downstream barriers (the neglect of upstream issues is of major importance in a decentralised state like Chad) and aligned with, not complementary to, immunization services strengthening activities. Further, both set of cash grants are blind to important recommendations such as the need to address barriers at the level of leadership and governance and at the level of the financing system and also about initiatives to promote community demand of vaccination services.

**Conclusion:**

In Chad slow vaccination progress is aggravated by several contextual barriers: the size of the country, the low population density, the nomadic nature of a significant part of its peoples, the recent civil war, associated with civil unrest and political instability and its geographical localization. In this situation it would be important to sustain downstream operations (the major focus of the ISS grant) while taking a long term view of the needs of the health system. The GAVI effectively supports downstream operations, but neglects the long term view.

## Introduction

Chad is a fragile state — with a harmonized score of 2.792 — with political and military instability right from the early years of independence [1] but the political situation is expected to remain stable for the near future [2]. Chad is one of ECCAS (Economic Community of Central African States) countries, a Regional economic community with six post-conflict, fragile, landlocked and sparsely populated countries. Its pivotal and strategic position makes it a potentially preferred transit zone between Regions of the continent. ECCAS has oil, mineral and mining resources, a huge agriculture and forestry potential and the continent's largest hydroelectric power prospects. It stands out as the African Region with the least infrastructural network for transport and energy, impacting negatively on social conditions and welfare [3]. Despite its potential wealth Chad has a GNP estimated at USD 600/inhabitant (2011) spending 7% of this on health. The UNDP ranks Chad as 183rd in terms of its human development index of 0.328 (http://hdrstats.undp.org/en/countries/profiles/map/, accessed on 08.01.2013). This paper tries to understand barriers to better vaccination coverage in the context of the GAVI support provided to the country.

**The health profile** The health profile of Chad is characterized by asymmetries between urban and rural areas and fixed and nomadic populations. The main health problems are malaria, tuberculosis, HIV/AIDS, diarrhoeal diseases, acute respiratory infections, malnutrition and maternal mortality [1, 4], compounded by frequent outbreaks of, mostly preventable, diseases. In 2011 the under-5 mortality rate was 203 per 1000 and the infant mortality was 126 per 1000. Progress towards the Millennium Development Goals has been slow [5]. Among other factors, this slow progress is probably related to the history of recent military conflicts, as under these conditions, governments spend more on defense and less on health and education [6].

**The health care system:** The organisation of Chad's health care follows a typical pyramidal system with three levels: the central Ministry of Public Health (MPH), responsible for the national strategic directions, the mobilisation and division of resources as well as checks on their use, guided by a national health policy (NHP) for 2007-2015, implemented through annual action plans of the regional health delegations and Health Districts (HD); the intermediary level, with 18 Regional Health Delegation (RHD), responsible for programming and support for implementation and monitoring of interventions; the peripheral level, the operational level, includes 68 HD, each with two levels: a 1st level made up of 865 Areas of Responsibility with at least one health centre planned or built in each (in 2006, 645 were operational with 684 health centres). The 2nd level includes a District hospital (6 out of 57 are both public and private) and a District Department. The HD is the hub for the implementation of the national health policy through a microplan. In their Areas of Responsibility the communities are organised into Health Committees (HC).

The shortage of nurses nationally has led the MPH to multiply the number of state and private schools with paramedic training across the country. These train technical health workers in two years and after they have worked for four years they may come back to the school and become a state registered nurse after one year of training.

**Vaccination coverage and vaccine preventable diseases:** Disease outbreaks are frequent in Chad, including of diseases such as dracunculiasis (during 2010) [7], but mostly of vaccine preventable diseases such as cholera [8, 9], bacterial meningitis [10–12], measles [13] and polio [14–19].

During a measles epidemic in 2004 - 2005, the capital city, N′Djamena, had immunization coverage of 33% in children under 5 years. Before the epidemic, supplemental immunization days (SID) “catch-up” campaigns had yet to be conducted in the country. The continuing high burden of preventable measles mortality during this epidemic resulted from poor access to appropriate treatment and the incomplete implementation of the WHO/UNICEF measles mortality-reduction strategy. Access to appropriate treatment was not provided free of charge from the beginning of the epidemic and most measles deaths occurred at home. Parents preferred to purchase treatment outside a health-care facility (e.g. market or pharmaceutical vendor), rather than to seek treatment there. Moreover, in facilities admitting patients with more severe measles, complications, excess workload and inappropriate case management of complications contributed to the mortality [20].

The downward trend in polio incidence in Chad was reverted since wild poliomyelitis virus was imported from Nigeria in 2001, with cases of poliomyelitis still occurring every year [10, 16–19, 21, 22]. The on-going efforts towards polio eradication lead to high primary care workloads, requiring an intense mobilization of health workers, limiting their availability for routine immunization activities [23]. Although no outbreak of yellow fever had been reported from Chad since the 1930-1950 campaigns, three cases were reported in 2009 [24].

**Vaccination services, coverage and data quality:** The health infrastructure is weak due to inadequate qualified medical staff and management skills at all levels, and a concentration of health services in urban areas [23, 25]. The service coverage needed to achieve the health related MDGs in Chad was estimated to require a fourfold increase in human resources [26]. Recurrent epidemics raise questions about the management of the expanded programme of immunization (EPI), questions which persist since the late 1990s [27].

**Figure 1:**
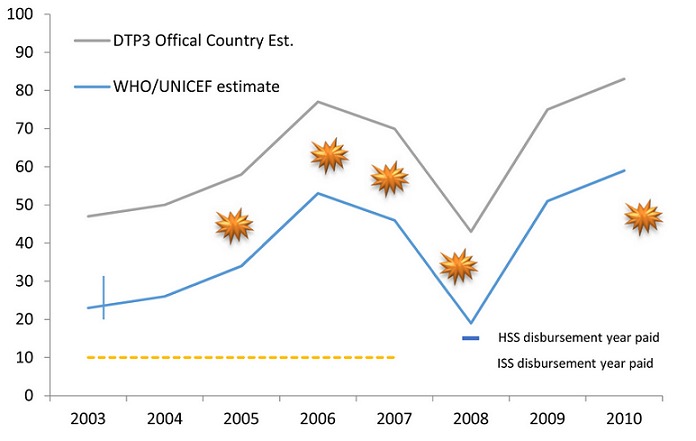
Chad DTP-3 coverage timeline

In 1985 EPI was a completely vertical programme. Since 1991 it was progressively integrated into district operations. As per the National Immunization Policy of 1999, the EPI's prime target has been to reduce vaccine related morbidity and mortality. The 1993 National Health Policy, revised in 1999, and again more recently, included vaccination services in the basic benefit package to be provided at first contact level [23]. With the support of GAVI, the government has decided to introduce new vaccines: Haemophilus influenzae b and Viral Hepatitis B vaccines in pentavalent form beginning 2008. Vaccination is provided in health facilities, in outreach services and during SID [27]. Even so, vaccination coverage remains low [21] (Figure 1).

**Figure 2:**
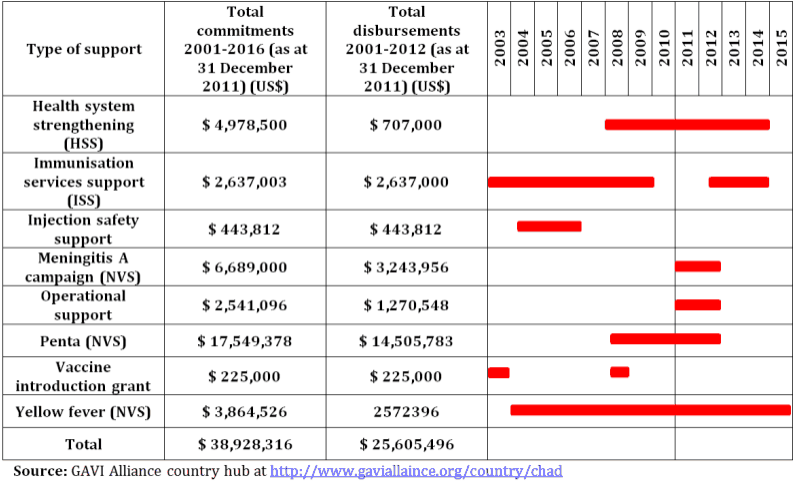
GAVI Alliance support to Chad

DTP-3 coverage has fluctuated over the past 10 years but increased in the last two. The country administrative coverage estimates mimic the trends of WHO/UNICEF estimates. No coverage survey has been conducted since 2003. According to WHO/UNICEF estimates, the DTP-3 coverage between 2003 and 2010 increased from 23% to 59% with an average yearly change of +4.5%. It dipped to a low of 19% in 2008, the year of the last GAVI disbursement, following years of political and military turmoil. Although it has not received any funds from GAVI since 2008, it has shown a steady and rapid increase since then (an average yearly change of +20%) (Figure 2) [28]

EPI and ante-natal care (ANC) attendances are strongly correlated. Residence, education level of mothers, partner's occupation and education and number of children under-5 are predictors of non-attendance at both ANC and EPI. Wealth index, ethnicity, religion and mother's occupation were not predictors. Women living in eastern and western regions of Chad were almost three times as likely not to attend either service compared to women in southern regions [29, 30].

**Supplemental Immunization Days:** SID is a strategy intended to accelerate eradication of poliomyelitis in countries where it is still endemic. In Chad, SID have repeatedly failed to reach a sufficient proportion of children in critical areas. Recent improvements in the quality of SID (2010) were the result of more effective governmental engagement and of increased external support [16]. After 30 SID in Chad and the inaccurate or false attribution of side-effects to polio vaccines, some groups persistently still refuse polio vaccination [21].

**Outreach vaccination services:** Outreach services are an essential component of service delivery in a sparsely populated country with a not insignificant nomadic population.

Outreach human vaccination services rarely exist for rural communities living far (>15 km) from the nearest health facility, which in Chad accounts for 40% of the rural population. Especially difficult is providing health services to nomadic groups, which also represent substantial numbers in Chad. Although now out of date, the most recent Chadian census identifies several nomadic groups which would have been seasonally located across the border in neighboring countries. Strategies of vaccinating mobile pastoralists on market days have been tested; however, children and women tend to stay in their camps, resulting in very low participation. In the nomadic camps, in 2000, livestock - compulsorily vaccinated by mobile veterinary teams - had a better coverage than women and children in those same camps[22].

Professionals from WHO, the Food and Agriculture Organization of the United Nations and others have proposed sharing of resources by public health and veterinary services to deliver health interventions at lower costs, thereby allowing for economies of scale. Chad is one of the few documented examples of joint implementation of this strategy. The Chadian Ministries of Public Health and of Livestock Production (hosting the veterinary services) implemented joint human and livestock vaccination campaigns. The capacity of existing mobile veterinary teams was extended for simultaneous vaccination of people and animals during at least 1 round for 10 of the 14 campaigns. The joint campaigns were organized in consultation with local health and veterinary personnel to avoid duplication of efforts and to make use of all existing personnel and infrastructure (cold chain and transportation). Before vaccination campaigns, trained community-based facilitators discussed with nomadic community members pictograms and short movies with health and veterinary messages. EPI provided human vaccines and consumables through the regional health administration, assessed continuously the number of vaccinated persons and was involved in the evaluation of the vaccination coverage. Pastoralist communities highly valued the combined approach that targeted the health of their family members and of their livestock. Increasingly, nomadic parents started visiting health centers with their children to seek vaccination services. Based on the positive outcomes of these pilot campaigns, Chadian public health and veterinary officials developed a common policy for child and livestock vaccination in pastoralist populations [22].

**Stocks and storage of vaccines**: Since the nineties there is record of difficulties with storage of vaccines and control of the cold chain at district level and of the variety of interventions implemented to improve the situation [27]. Difficulties in purchasing vaccines through the governmental budget lead to recurrent stock-outs of vaccines [23]. The stock-outs are compounded by vaccine store management issues, as identified during the last Effective Vaccine Store Management (EVSM)/Vaccine Management Assessment (VMA), conducted in 2005, which recommended a number of interventions, namely: (i) displaying in the different stores of the contact details of the relevant personnel for emergencies; (ii) strengthening temperature monitoring; (iii) fencing EPI sites to secure storage of vaccines; (iv) building a controlling office in a place convenient for storage control; (v) developing a plan of preventive maintenance for buildings and equipment; (vi) ensuring the back up of electronic data at least once a week; (vii) demanding the submission of freeze indicators. A follow up VMA planned for 2009 did not take place.

**Funding of the health system and of EPI:** Total health expenditure per capita in 2009 was USD41.80, representing an increase from the USD18.40 of 2002. General government health expenditure as a percentage of total health expenditure for the same period increased from 34% to 51%. Official development assistance (ODA) for health to Chad represented about 2.9 USD per capita and 5.9% of the total ODA to the country in 2009. In 2009, the five largest sources of ODA for health (representing 68% of all ODA for Health) were the governments of the France (17%), the European Commission (15%), GAVI (13%), UNFPA (12%) and GFTAM (11%) [31].

### Cash-based support from the GAVI Alliance

GAVI has been assisting the Government of Chad since 2002 with: new and underused vaccine support (NVS); immunization services support (ISS); injection safety support (INS) (ended in 2004); and health system's strengthening support (HSS) (Table 1). GAVI HSS funds represent 0.6% of government health expenditure, 0.3% of total health expenditure and 1.8% of ODA [32].

**Table 1:** GAVI Alliance disbursements for ISS and HSS (USD)

Countries	GAVI window	Commitment	Start disbursement	End disbursement	total commitment	Disbursement as of July 2011	Disbursement per inhabitant per year
Chad	ISS	2003-2007	2003	2007	2,637,000	2,637,000	0.03
	HSS	2008-2012	2008	2008	4,978,500	707,000	0.02
	Total				7,615,500	3,344,000	0.04

ISS: Immunization Services Support; HSS: Health System's Strengthening; GAVI: The Global Alliance for Vaccines and Immunization; MOH: Ministry of Health; USD: United States Dollars.

### Study objectives

Considering the vaccination situation and the relative importance of the GAVI Alliance cash based funds in sustaining vaccination services, it was decided to document the relative importance of the GAVI-cash based funds related factors that might have influenced negatively the immunization coverage in Chad.

## Methods

The paper reports on a modified Delphi study and on document analysis.

**Modified Delphi study:** The Delphi technique is group facilitation, iterative, multistage procedure designed to convert individual opinions into a group consensus [33–35]. It has been applied to many health research questions, not infrequently with operational modifications [42, 43]. A modified four rounds Delphi study, on two consecutive days, of the negative influences on the immunization coverage was carried during 24-26 October 2011. Qualitative data was concurrently collected throughout the different Delphi rounds, mostly in an unstructured way. During Round 1 the panelists were asked to independently quantify the relative contribution of the following 5 tentative causes expressed as a percentage (the total of the 5 causes should add up to 100%).

Design failure (inappropriate design of the HSS/ISS proposal) Implementation failure (bad implementation due to capacity constraints, changes in plan, etc.), Inadequate governance of the grant (administrative delays, leakage, etc.), Insufficient amount of the funds made available through the HSS/ISS proposal and or lack of complementary funding, External factors, exogenous to the health sector (civil war, disasters; etc.).

Country work was carried out in N'djamena by an external WHO consultant with support of the national WHO office and the MPH who assisted with recruitment of local experts to serve on the panel (Table 2) and who met together on the second day of the study for rounds 2-4. The consultant facilitated the rounds and associated discussions, calculated means, medians, modes ranges, for Round 1 results and asked the panellists for re-estimates for three further rounds, confronting them with the summary results of the previous round, in order to move them, without coercion, towards a consensus position on the estimates. Consensus was considered when the distance between the median and the average was less than 2% or for any range of values that included at least 75% of the replies (Table 2).

**Table 2:** Panellists, calendar and procedures followed

Initial contact	15 invited to participate
Round 1	10 participated in email Round on the 25^th^ of October
Round 2	11 participated in Rounds 2,3,4 on the 26^th^ of October
Round 3	
Round 4	
Origin of participants	2 from WHO, 1 from UNICEF, 1 from European Union and the rest from the MPH

**UNICEF:** The United Nations Children's Fund; MPH: Ministry of Public Health.

**Document analysis:** Document analysis is a recognized qualitative research methos, as such adopted to scrutinise the study documents [38]. The documents were included if found in the GAVI Alliance′s country hub for Chad in March 2012. The documents analysed were: ISS annual progress report (APR) from 2005 to 2009; the 2008 HSS grant submissions; the 2010 HSS APR.

The analysis conducted was similar to the one described by Goeman et al (2010) [39] and it was based on WHO's health system building block categories - service delivery, health workforce, health information systems, logistics (medical products, vaccines and technology), financing and leadership & governance [40]. This categorization was extended to include a classification of activities at either the operational (downstream activities) or the systemic (upstream) levels. Downstream activities were defined as those that one would reasonably assume exist at the district healthcare system level or below and do not involve comprehensive change at a higher, systemic level. Upstream interventions were defined as those taking place at and/or involving change (action or resources) at a level higher than the district healthcare system level. Where an intervention could be considered systemic and operational, it was classified as systemic. No one building block category was considered to be uniquely systemic or operational. To these we added an additional category, community orientation [39, 41].

## Results

The results are first presented for the modified Delphi and then for the document analysis.

Understanding failing vaccination efforts using a modified Delphi approach.

For the Delphi, the quantitative results are presented before the qualitative results.

**Quantitative findings:** After the four rounds, according to the panelists:

Implementation problems, explain on average 46% of the failure to perform better with regard to vaccination. It was considered the main bottleneck;

- Governance occupied second position with an average of 23%;
- Conceptualization of the projects was the third issue with an average of 14%;
- Scarcity of funds was fourth with an average of 9%; and
- Extrinsic factors were the fifth factor with an average of 7%.

After applying the different definitions of consensus to the data available for the last round a range of possible consensus values was obtained. From Table 3, the lowest value of the narrowest range of values for a specific factor was adopted as the consensus estimate for the country (Table 4).

**Table 3:** Range of consensus values according to different definitions of consensus

Definition of consensus	Range of consensus values (%) according to different definitions				
Factors explaining vaccination performance	1	2	3	4	5
Difference between mean and median (or vice-versa) ≤ 2		45-46	23-25		5-7
any range of values that included at least 75% of the replies	5-20	40-60	20-35	5-25	5
Note: Design failure (inappropriate design of the HSS/ISS proposal) Implementation failure (bad implementation due to capacity constraints, changes in plan, etc.) Inadequate governance of the grant (administrative delays, leakage, etc.) Insufficient size of the funds made available through the HSS/ISS proposal and or lack of complementary funding External factors, exogenous to the health sector (civil war, disasters; etc.)					

**Table 4:** Consensus values (%) adopted

Causes of unsatisfactory vaccination coverage studied	Consensus contribution
a problem of implementation	At least 45%
a problem of governance	At least 23%
a problem of scarce funds	At least 5%
extrinsic problems	At least 5%
a problem of conceptualization	At least 5%

The consensus on Table 4 confirms the prominence of implementation and governance factors and ranks equally the three other factors.

**Qualitative findings:** Table 5 summarises the qualitative data collected during discussions taking place while the Delphi Rounds were conducted.

**Table 5:** Qualitative data from rounds 2 to 4: key reasons for the major factors of poor performance

Domains of analysis	Reasons given
**Conceptualization**	Difficult to expect any measurable impact on a HSS program of 3 years
**Implementation**	Resources for ISS do not reach the regions
**Governance**	Steering Committee not functioning adequately Incompatibility between public service administration and GAVI administrative procedures Turnover of senior personnel in the MOH – new personnel did not understand the spirit of the HSS project
**Level of funding**	Level of funding considered adequate
**Human resources**	Turnover of senior personnel is a major bottleneck
**Community level**	It was not addressed
**External factors**	Political and military instability

HSS: Health System's Strengthening;GAVI: The Global Alliance for Vaccines and Immunization; MOH: Ministry of Health.

**Recommendations:** The final discussion on consensus recommendations to the Board of the GAVI Alliance flowed from the qualitative data (Table 6).

**Table 6:** Recommendations to the GAVI Board by the Delphi panellists

Domains for GAVI support	Recommendations
**Conceptualization of the proposals**	The HSS proposal must be more realistic for the time horizon of the subvention – it is not possible to expect impact on the health system, but rather on the health system building blocks.
**Leadership and governance**	Improve leadership capacity and management skills: strategic and “day to day” GAVI and the MOH must agree on mechanisms to stabilize and retain personnel responsible for the management of the project Improve communication between GAVI and MOH Establish adequate administrative structures for the GAVI funds
**Implementation**	Will be more successful if attention is given to governance and management capacities, including capacities at decentralized levels of the health system.
**Financing**	Improve skills to administer cash funds.

HSS: Health System's Strengthening; GAVI: The Global Alliance for Vaccines and Immunization; MOH: Ministry of Health.

### Analysis of grant documents and reports

The focus of the barriers and of the activities of the ISS grant APR has been downstream. The major groups of barriers reported are associated with community orientation, logistics and health information systems and monitoring and evaluation. The activities reported, which focus typically on downstream interventions, are associated mostly with service provision, logistics and the health workforce (generally training). Of the 90 activities reported only 1 focused on the financing system (Table 7).

**Table 7:** Number of barriers identified and of activities proposed in the GAVI cash grants according to building blocks and to their upstream/downstream nature

Health system's building block and community orientation	ISS cash support - annual progress reports 2005-20010				HSS cash support							
					Grant application				Annual progress report 2010			
	Barriers identified		Activities reported		Barriers		Activities proposed		Barriers		Activities	
	Upstream	Downstream	Upstream	Downstream	Upstream	Downstream	Upstream	Downstream	Upstream	Downstream	Upstream	Downstream
Leadership and governance	1	0	3	9	2	4	0	3	4	0	2	1
Health workforce	2	0	3	14	3	10	0	7	0	1	0	7
Logistics	4	11	3	15	0	2	0	2	0	3	0	2
Service provision	0	9	1	25	0	3	0	0	0	2	0	0
Health information systems and monitoring and evaluation	1	11	4	2	0	3	1	0	0	0	0	0
Financing system	2	2	1	0	0	2	0	0	3	1	0	0
Community orientation	7	10	4	6	0	5	0	0	0	0	0	0
Total	17	43	19	71	5	29	1	12	7	7	2	10

The HSS cash-grant application is ambitious in the identification of barriers (all, but 1, downstream) with the interventions focused almost totally (all but 1 activity) downstream (mostly health workforce related, usually training), remaining blind to upstream issues (of major importance in a decentralised state like Chad), to the financing system, to service provision (this might be appropriate considering the focus of the ISS grant), community orientation and health information systems and monitoring and evaluation (Table 7).

The first and only APR of the HSS analysed has a balanced view of upstream/downstream nature of the barriers, putting on the map barriers associated with the financing system which were ignored in the HSS grant application. The activities reported were, as expected considering the nature of the grant application, mostly downstream and associated with the health workforce (mostly training). As for the grant application, the APR activities are blind to the financing system, to service provision (this might be appropriate considering the focus of the ISS grant), community orientation and health information systems and monitoring and evaluation (Table 7).

## Discussion

Most conflict-affected countries rely heavily upon international aid and humanitarian assistance for basic service provision as internal state capacities are limited. Conflict-affected low-income countries have worse development indicators than non-conflict-affected low-income countries. Conflict increases vulnerability to poor health as: (i) the health service infrastructure and human resources can be severely depleted; (ii) access to health services, information and supplies is reduced; (iii) exposure to sexual violence increases; and (iv) impoverishment and related risk-taking behaviour rises. Despite this evidence, the health sector has historically received insufficient attention in conflict-affected countries [42] and the operational focus of the attention does little to strengthen health systems and improve sustainability. The situation described for Chad by the panelists illustrates this conflict situation panelists.

In Chad slow vaccination progress is aggravated by several contextual barriers: the size of the country, the low population density, the nomadic nature of a significant part of its peoples, the recent civil war, associated with civil unrest and political instability and its geographical localization, bordering countries, such as Nigeria, which are foci of imported diseases into Chad. One consequence of this situation, as pointed out by the Delphi panellists, is that resources tend not to reach remote areas and nomadic populations. Chad′s experience of combining human vaccination services with veterinary vaccination services point to innovative solutions that should be scaled up [22].

In this situation it is important to sustain downstream operations (the major focus of the ISS grant) while taking a long term view of the needs of the health system. As none of the grants takes this long term view, the HSS grant assumes a supplementary rather than a complementary role in relation to the ISS grants, while both remain blind to barriers at the level of leadership and governance and at the level of the financing system. Activities under HSS fund more of the same already funded by ISS. The blindness of GAVI HSS proposals to the need to address issues related to the financing system has been identified in a previous study [39].

The difficulty in considering interventions targeting sustainable results with narrow-time horizons, acknowledged by the Delphi panellists, echoes Jeffrey Sachs statement that “it's not a year or two of help that we need, but it's 20 years of help” (cited in Hardon and Blume 2005) [43]. This long term view,, once adopted, would align the philosophy of funding with current debates on the sustainability of aid for development subsequent on Busan's 4th High Level Forum on Aid Effectiveness, held at the end of 2011 [46]. It should lead to a broader systemic view, capturing intersectoral issues related to leadership and governance, public service regulations and to the public financing sub-system.

HR issues are not insignificant. These include quantitative and qualitative shortages, operational weakness of the teams on the ground and inadequate cadres of managers. The Delphi panellists singled out the high turnover of senior managers as a major bottleneck and pointed to the inadequate administrative and financial management systems, usually performing better centrally than at the periphery, a problem identified in the barriers detected during the document analysis but not reflected in the actions proposed or carried out. The focus on downstream training activities falls short of the needed interventions recognised in the literature [38] and pointed out by the Delphi panel (Table 5).

All the documents analysed were blind to and the Delphi panel was silent about initiatives to promote community demand of vaccination services. Even more surprising in a country where as a result of the inaccurate or false attribution of side-effects to polio vaccines, some groups persistently still refuse polio vaccination [21]. This neglect may reflect that the effectiveness and cost-effectiveness of efforts to increase demand is still uncertain [48].

The discrepancies between DTP3 official country estimates and WHO/UNICEF are recognized: as for other countries, in Chad government estimates usually mimic WHO-UNICEF estimates at a higher level [49, 50]. Data problems were also identified in several data quality assessments: absence of written official regulations for data collection or back up procedures, to save data; a stock ledger sheet for entry and exit of syringes and vaccines was not always available or adequately filled; some districts did not systematically register the drop-out rate; absence of registers on timeliness of reports; there was no system in place to monitor vaccine wastage and adverse effects; health units are generally not aware of new births in their target area, despite a simple reporting system used by traditional birth assistants and village health agents; in some health units storage practices were deficient, documents could not easily be retrieved and were not classified in chronological order or could not be found [23]. Therefore, it is quite inappropriate that health information issues are not emphasized in most APR and proposals and not acknowledged by the Delphi panel. This is even more important because if district data are to be used in monitoring and planning immunization programmes, as intended by decentralization, heterogeneity in their validity must be reduced [51].

Lack of maintenance and renovation of cold chain, transport and computer equipment is another significant problem interfering with vaccination performance.

## Conclusion

Most of the activities anticipated under the GAVI health system's strengthening proposal are activities targeting downstream barriers (the neglect of upstream issues is of major importance in a decentralised fragile state like Chad) and aligned with, not complementary to, immunization services strengthening activities. This probably reflects the incapacity of weak governance systems addressing downstream issued within a common national governance framework, partially reflecting the level of decentralization and the lack of responsiveness to its very specific governance issues [52]. Further, both set of cash grants are blind to important recommendations such as the need to address barriers at the level of leadership and governance and at the level of the financing system and also about initiatives to promote community demand of vaccination services. The Delphi panel ignores this last issue, in a country where popular opposition to vaccination is not insignificant.
